# Gene up-regulation in response to predator kairomones in the water flea, *Daphnia pulex*

**DOI:** 10.1186/1471-213X-10-45

**Published:** 2010-04-30

**Authors:** Hitoshi Miyakawa, Maki Imai, Naoki Sugimoto, Yuki Ishikawa, Asano Ishikawa, Hidehiko Ishigaki, Yasukazu Okada, Satoshi Miyazaki, Shigeyuki Koshikawa, Richard Cornette, Toru Miura

**Affiliations:** 1 Graduate School of Environmental Science, Hokkaido University, Sapporo, Hokkaido 060-0810, Japan; 2 Graduate School of Environmental Science, Okayama University, Okayama, Okayama 700-8530, Japan; 3 Graduate School of Veterinary Medicine, Hokkaido University, Sapporo, Hokkaido 060-0818, Japan; 4 Laboratory of Molecular Biology, University of Wisconsin-Madison, Madison, Wisconsin 53706, USA; 5 Anhydrobiosis Research Unit, National Institute of Agrobiological Sciences, Tsukuba, Ibaraki 305-8634, Japan

## Abstract

**Background:**

Numerous cases of predator-induced polyphenisms, in which alternate phenotypes are produced in response to extrinsic stimuli, have been reported in aquatic taxa to date. The genus *Daphnia *(Branchiopoda, Cladocera) provides a model experimental system for the study of the developmental mechanisms and evolutionary processes associated with predator-induced polyphenisms. In *D. pulex*, juveniles form neckteeth in response to predatory kairomones released by *Chaoborus *larvae (Insecta, Diptera).

**Results:**

Previous studies suggest that the timing of the sensitivity to kairomones in *D. pulex *can generally be divided into the embryonic and postembryonic developmental periods. We therefore examined which of the genes in the embryonic and first-instar juvenile stages exhibit different expression levels in the presence or absence of predator kairomones. Employing a candidate gene approach and identifying differentially-expressed genes revealed that the morphogenetic factors, *Hox3*, *extradenticle *and *escargot*, were up-regulated by kairomones in the postembryonic stage and may potentially be responsible for defense morph formation. In addition, the juvenile hormone pathway genes, *JHAMT *and *Met*, and the insulin signaling pathway genes, *InR *and *IRS-1*, were up-regulated in the first-instar stage. It is well known that these hormonal pathways are involved in physiological regulation following morphogenesis in many insect species. During the embryonic stage when morphotypes were determined, one of the novel genes identified by differential display was up-regulated, suggesting that this gene may be related to morphotype determination. Biological functions of the up-regulated genes are discussed in the context of defense morph formation.

**Conclusions:**

It is suggested that, following the reception of kairomone signals, the identified genes are involved in a series of defensive phenotypic alterations and the production of a defensive phenotype.

## Background

**Figure 1:**
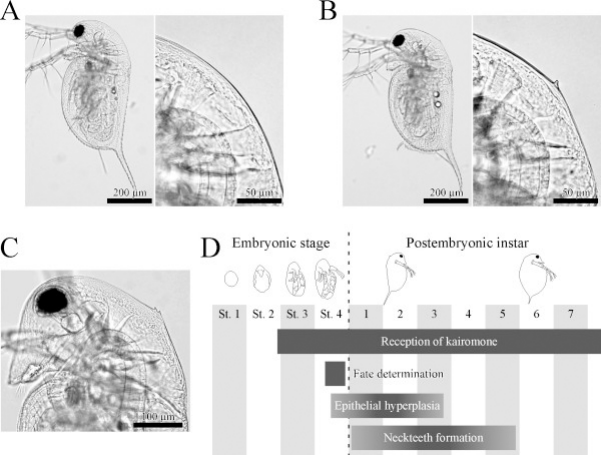
**Chaoborus-induced defense morph formation in *D. pulex***. *Chaoborus*-induced defense morph formation in *D. pulex*. (A) A normal first-instar juvenile of *Daphnia pulex *(magnified view of occipital region: right). (B) A first-instar juvenile bearing neckteeth (magnified view of neckteeth: right). (C) A second-instar juvenile bearing larger neckteeth with two spines. (D) Schematic diagram of the developmental events in the process of the defense morph formation in *D. pulex *(based on [6]).

The ability to modulate development in the presence of predators is referred as "inducible defense" or "predator-induced polyphenism" [1]. Of the examples reported to date, the freshwater microcrustacean genus *Daphnia*, commonly called the waterflea, is considered to be a model case for elucidating the ecological and developmental underpinnings of this process [2]. *D. pulex *produces structures referred to as neckteeth on its head, primarily in the earlier instars (first to third instar), in the presence of predatory phantom midges (*Chaoborus *larvae) (Figure 1A, B, C). It is considered that this morphological change in daphnids occurs in response to being exposed to chemical cues referred to as "kairomones" released by predators [3]. Neckteeth effectively decrease the risk of predation because it is difficult for predators to capture *Daphnia *juveniles that have this outgrowth [3]. Several investigations have shown that *D. pulex *exhibits sensitivity to *Chaoborus *kairomones during embryonic development [4,5]. In addition, the additive effects of these chemical cues and sensitive phases of *D. pulex *embryos and juveniles have been elucidated [6].

Predator-induced defenses most commonly involve morphological and behavioral changes, which allow them to escape from predators efficiently and contribute positively toward the overall ecological success of *Daphnia *[3,7]. The morphological changes in *Daphnia *that occur in response to the existence of predators are considered to be among the most remarkable examples of polyphenism, particularly since individuals with the same genetic background can express a variety of phenotypes in response to different environmental cues. During postembryonic development, neckteeth have been observed in the first instar, although the rate of induction in this instar is generally weak [6]. Induction rates were generally stronger in the second and third instars, before becoming weaker again in the fourth instar; neckteeth have not been reported in *Daphnia *after the fifth instar [8,9]. It is thought that *Daphnia *are most sensitive to kairomone signals during embryonic development [4], but exposure to the chemical cues after the first-instar juvenile stage is also required for the maintenance of these structures [6]. The morphogenesis of neckteeth appears to start during embryogenesis [10]. Although the developmental mechanisms by which multiple alternative phenotypes are produced have attracted considerable interest [11-19], little is known about the molecular basis underlying the developmental regulation of defense morph formation.

The waterflea *Daphnia *has come to be widely used as a model animal for a variety of reasons [20]. Specifically, since *Daphnia *is a keystone species in ponds and lakes [21], it has been used for decades as a standard organism for toxicity testing and its toxicological responses to environmental pollutants are well characterized [22,23]. Furthermore, *Daphnia pulex *is the first crustacean to have had its genome sequenced [23,24], and these new genomic data are likely to facilitate studies in the wide variety of research disciplines that employ *Daphnia *(e.g. [25,26]). Indeed, the availability of the draft *Daphnia pulex *genome sequence assembly and annotation v1.1 promotes the development of a new model system for ecological and evolutionary genomics [2,23,27].

In this study, several experiments using molecular tools were designed to elucidate the molecular mechanisms underlying defense morph formation in response to predator kairomones. Our previous study, which established the defense-morph induction by the exposure of dissected embryos to kairomone, revealed that kairomone reception during the late-embryonic stage (stages 3 and 4; the embryonic development is divided into 4 stages) is required for the future development of neckteeth, although the defensive traits only appear in postembryonic instars, particularly the second- and third-instars (Figure 1C) [6]. These findings suggest that, downstream of kairomone reception, the mechanisms responsible for fate determination and neckteeth development are activated in stage-4 embryos and first-instar juveniles, respectively (Figure 1D). In *Daphnia*, kairomone reception is thought to initiate a series of biological reactions; neuronal signals which are converted into endocrine signals that then subsequently induce changes in the expressions of morphogenetic factors and result in defense morph formation. The occurrence of these reactions has been inferred by recent studies on polyphenic development in insects [10,28-30]. Based on this working hypothesis, we searched the *Daphnia *genome database (wFleaBase) for genes affecting endocrine, morphogenetic and neuronal regulations, which are considered to be involved in the defense-morph formation. Then, using real-time quantitative RT-PCR, we sought to determine whether the observed mRNA expression levels of these gene sequences changed in response to kairomone treatment in stage-4 embryos and the first-instar juveniles. Furthermore, to identify any unknown genes involved in these processes, we also screened genes that had been differentially expressed in response to kairomone exposure using a differential display method (see Methods). Based on these results, the functions of these genes are discussed in terms of kairomone reception and defense morph formation.

## Results and Discussion

### Selection of candidate genes

In *D. pulex *defense morph formation, it is thought that morphogenetic factors such as those that have been identified in many arthropods are expressed downstream of physiological regulation [30,31]. The following 31 candidate genes were identified in the *D. pulex *genome: Hox genes [*labial *(*lb*), *proboscipedia *(*pb*), *Hox3*, *Deformed *(*Dfd*), *Sex combs reduced *(*Scr*), *Antennapedia *(*Antp*), *Ultrabithorax *(*Ubx*), *abdominal-A *(*abd-A*) and *Abdominal-B *(*Abd-B*)], morphogenetic genes [*Distal-less *(*Dll*), *aristaless *(*al*), *homothorax *(*hth*), *dachshund *(*dac*), *extradenticle *(*exd*), *escargot *(*esg*), *teashirt *(*tsh*), *epidermal growth factor receptor *(*EGFR1*, *2*), *spitz *(*spi*), *decapentaplegic *(*dpp*), *wingless *(*wg*) and *hedgehog *(*hh*)], endocrine genes [*juvenile hormone acid methyltransferase *(*JHAMT*), *Methoprene-tolerant *(*Met*), *ultraspiracle *(*USP*), *ecdysone receptor *(*EcR*), *insulin-like receptor *(*InR*), *insulin receptor substrate-1 *(*IRS-1*) and *forkhead box O *(*FOXO*)] and neuronal genes [*tyramine beta-monooxygenase *(*TBM*) and *dopamine beta-monooxygenase *(*DBM*)]. BLASTX searches http://blast.ncbi.nlm.nih.gov/Blast.cgi confirmed that the predicted *D. pulex *sequences are homologues of the candidate genes (Table 1, Additional file 1).

**Table 1:** Expression profiles of investigated candidate genes

		Relative expression^a^	
		
Candidate gene (abbreviation)	*Daphnia pulex *gene ID	Embryo	Juvenile
*Hox gene*			
labial (lb)	Dappu-36672 Dappu-97497	1.35	**1.598**
proboscipedia (pb)	Dappu-44300 Dappu-97500	1.433	1.276
Hox3	Dappu-9456	1.312	**1.9**
Deformed (Dfd)	Dappu-44270 Dappu-97505	1.35	**1.541**
Sex combs reduced (Scr)	Dappu-37195 Dappu-44375 Dappu-97506	1.199	**1.55**
Antennapedia (Antp)	Dappu-44334 Dappu-236216	1.254	1.248
Ultrabithorax (Ubx)	Dappu-9277 Dappu-221891	1.253	**1.601**
Abdominal-A (Abd-A)	Dappu-29076	Not amplified	
Abdominal-B (Abd-B)	Dappu-29045 Dappu-97516	1.235	**1.82**
			
*Morphogenetic gene*			
Distal-less (Dll)	Dappu-9287	1.365	1.2
aristaless (al)	Dappu-37455	1.212	**1.597**
homothorax (hth)	Dappu-4560 Dappu-5009	1.1	**1.539**
dachshund (dac)	Dappu-94521 Dappu-94522 Dappu-232746 Dappu-232754	1.05	**1.591**
extradenticle (exd)	Dappu-219790	1.128	**2.131**
escargot (esg)	Dappu-50534	0.7	**1.951**
teashirt (tsh)	Dappu-99118	1.276	1.377
epidermal growth factor receptor 1 (EGFR1)	Not predicted	1.193	**1.505**
epidermal growth factor receptor 2 (EGFR2)	Dappu-9119 Dappu-53919	1.174	**1.67**
spitz (spi)	Dappu-271340	1.101	1.181
decapentaplegic (dpp)	Dappu-40449	0.992	1.471
wingless (wg)	Dappu-290640	1.306	**1.616**
hedgehog (hh)	Dappu-290571	1.122	**1.579**
			
*Endocrine gene*			
juvenile hormone acid methyltransferase (JHAMT)	Dappu-300180	0.365	**2.707**
Methoprene-tolerant (Met)	Dappu-247693	0.943	**1.584**
ultraspiracle (USP)	Dappu-219609	0.775	1.354
ecdysone receptor (EcR)	Dappu-319648	0.736	1.158
insulin-like receptor (InR)	Dappu-270048	0.84	**1.956**
insulin receptor substrate-1 (IRS-1)	Dappu-52188 Dappu-304473	0.825	**1.81**
forkhead box O (FOXO)	Dappu-109005	Not amplified	
			
*Neuronal gene*			
tyramine beta-monooxygenase (TBM)	Dappu-1839	1.191	**3.897**
dopamine beta-monooxygenase (DBM)	Dappu-62540	1.14	**1.686**

^a ^Expression levels in the presence of the kairomones are displayed relative to control medium, measured by first real-time quantitative PCR. Values in bold indicate more than 1.5-fold increase.

See additional file 1 placing annotations of these genes.

### Expression profiles of candidate genes

The relative expression levels of the candidate genes were quantified using real-time quantitative RT-PCR to examine whether these genes were differentially expressed after exposure to the predator kairomone in the embryonic stage (stage 4) and the postembryonic instar (first-instar). 18S ribosomal RNA, *actin*, *glyceraldehyde-3-phosphate dehydrogenase *(*GAPDH*) genes were tested as reference genes for real-time qPCR. Since *GAPDH *was considered to be the most stable gene and its expression levels were closest to those of the candidate genes (data not shown), *GAPDH *was used as the reference gene. This result was consistent with a previous report of expression stability in *D. magna *following exposure to the drug, ibuprofen [32].

Real-time qPCR revealed that 21 of the 31 candidate genes were up-regulated in the presence of kairomones in first-instar juveniles. Conversely, none of the candidate genes were up-regulated by more than 1.5-fold at the embryonic stage 4 (Table 1, Additional file 1). Two genes (*abd-A *and *FOXO*) were not amplified by PCR, probably because the designed primer sites were inappropriate. Since a number of traits in first-instar juveniles change in response to defense morph formation (e.g. crest epithelial hyperplasia), the candidate genes were thought to be up-regulated in the preceding developmental stage (i.e. embryonic stage 4), although the expected results were not obtained. It is therefore suggested that the up-regulated genes are involved in neckteeth development, which is most pronounced at the second- and third-instar [6]. Furthermore, these results show that the candidate gene approach using a *Daphnia *genome database can be used for the analysis of the molecular mechanisms responsible for the defense morph formation.

**Figure 2:**
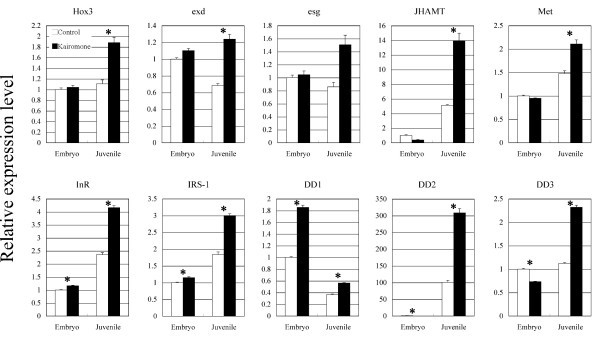
**Relative expression levels of *JHAMT*, *Met*, *InR*, *IRS-1*, *Hox3*, *exd*, *esg*, *DD1*, *DD2 *and *DD3 *in stage 4 embryos and the first-instar juveniles treated with kairomone and control media, analyzed by real-time quantitative PCR**. *DD1 *showed higher levels of expression after exposure to kairomone medium (black) than in control medium (white) during embryonic development. Other genes showed higher levels of expression after exposure to the kairomone medium than in the control during juvenile development. Y-axes indicate relative expression levels normalized by comparison with GAPDH expression (internal control gene). Technical triplicates were performed for all reactions. Bars indicate standard errors. Asterisks indicate significant differences (P < 0.05, based on [57]).

To further clarify differences in the expression of the six candidate genes showing the most marked up-regulation (*TBM*, *JHAMT*, *exd*, *InR*, *esg*, *Hox3*) and the two gene candidates thought to be associated with *JHAMT *(*Met*) and *InR *(*IRS-1*), we reperformed the real-time qPCR using biological replicates to analyze the detailed expression profiles of kairomone-responsive genes whose functions were suspected of being involved in defense morph formation (Figure 2). Unfortunately, since the level of expression of *TBM *varied between trials (possibly due to low expression level), we excluded *TBM *from further analyses.

### Endocrine genes (*JHAMT, Met, InR and IRS-1*)

Compared to when no kairomones were present, the expression levels of *JHAMT*, *Met*, *InR *and *IRS-1 *in first-instar juveniles exposed to kairomones increased by approximately 2.5-, 1.5-, 1.8- and 1.6-fold, respectively (Figure 2). *JHAMT *encodes the methyltransferase that mediates the final step of juvenile hormone synthesis [33], *Met *encodes a candidate receptor for juvenile hormone [34,35], *InR *encodes an insulin/insulin-like growth factor receptor, and *IRS-1 *encodes a downstream element that interacts directly with InR [36].

In addition to polyphenism [37-39], juvenile hormones (juvenoids) constitute a group of acyclic sesquiterpenoids that are key hormones in the regulation of a variety of physiological regulations in insect development and morphogenesis [28,40]. In crustaceans, methyl farnesoate (MF) is known to act as a juvenile hormone and plays important roles in the regulation of development [41]. For example, male production can be induced in female daphnids treated with MF [42]; however, little is known about other functions of MF in *D. pulex*. In addition to JH, the insulin-signaling pathway in many animals is also important for the regulation of a variety of developmental processes, including body-size and allometry controls [30,36,43,44]. It has been suggested that the crosstalk between the JH and insulin-signaling pathways is responsible for the expression of morphogenetic factors in the development of beetle horns [30]. Thus, it appears that physiological regulation by these endocrine factors may induce the expression of morphogenetic genes resulting in neckteeth formation in *D. pulex*.

### Morphogenetic genes (*Hox3*, *extradenticle*, *escargot*)

Compared to conditions without kairomones, the expression levels of *Hox3*, *extradenticle *and *escargot *in first-instar juveniles increased by approximately 1.7-, 1.9- and 1.8-fold, respectively, in the presence of kairomones (Figure 2). *Hox3 *is a member of the Hox cluster and appears to have a typical Hox-like role in the centipede, whereas the insect *Hox3 *ortholog, *zerknüllt *(*zen*), has lost the function of specifying segmental identity during embryogenesis [31,45-47]. Although little is known about the functions of crustacean *Hox3*, expression in *D. pulex *has been reported in the nuchal area (where the neckteeth subsequently form) and in the mandibular mesoderm during the early- and mid-embryonic stages, respectively [48], suggesting a possible role in establishing the position of neckteeth development. Furthermore, although *Daphnia *neckteeth cannot be considered to be homologous to appendages, it is possible that the molecular mechanisms for appendage development are co-opted for neckteeth development.

*Exd *and *esg *respectively encode a homeobox transcription factor and a zinc finger transcription factor, and both are known to determine the proximal segmental identity of appendages (coxa and trochanter) in *Drosophila melanogaster *[49]. Furthermore, up-regulation of *dac*, a known selector gene for the femur and tibia in *D. melanogaster*, and *Dll*, which defines tarsus and pretarsus, did not produce as conspicuous a response as *exd *and *esg *in first-instar juveniles (1.6- and 1.2-fold, respectively) (Table 1, Additional file 1). This evidence showed that the genes responsible for the determination of proximal appendages were up-regulated in juveniles with neckteeth, implies that these genes might be co-opted for neckteeth formation. However, our results also showed that the expression level of *al*, a known selector gene for the most distal region of appendage [49], was also higher (Table 1, Additional file 1). This is probably because, in *Drosophila*, *al *is also expressed in the proximal regions of appendages [49]. It has recently been reported that *Dll *and *al *are both involved in the development of beetle horns, which are not homologous to appendages [29]. This is similar to the situation in the *Daphnia *neckteeth formation, except that the co-opted regions of appendages are different (proximal or distal). Indeed, further analyses of this hypothesis will provide us with insights, not only into defense morph formation in *Daphnia*, but also into the evolution of appendage morphology in arthropods.

### Exploring novel genes by differential display

Next, differential display was performed to identify any novel genes that were related to neckteeth formation, but which were not included in the candidate gene approach. As a result, we obtained 22 fragments exhibiting differential expressions in response to kairomone exposure. To refine these results further, BLASTN searches were used to compare these fragments against wFleaBase and their coding sequences were predicted using a gene prediction software joined to wFleabase (Gnomon, Dappu v1.1 gene models, SNAP gene predictor). For the single fragment for which the functional sequence could not be predicted by the gene predictor, the full sequence was determined by rapid amplification of cDNA ends (RACE)-PCR. Of these 22 sequences, we performed real-time quantitative RT-PCR to confirm their responsiveness as described for the candidate genes above. As a result, three genes (*DD1*, *DD2 *and *DD3*) showed marked up-regulation in response to kairomone exposure (Figure 2, Table 2, Additional file 2).

**Table 2:** Expression profiles of the genes obtained by differential display (DD)

	Relative expression^a^	
	
Sequence name	Embryo	Juvenile
DD1	1.35	**1.598**
DD2	1.433	1.276
DD3	1.312	**1.9**

^a ^Expression levels in the presence of the kairomones are displayed relative to control medium, measured by first real-time quantitative PCR. Values in bold indicate more than 1.5-fold increase.

See additional file 2 placing annotations of these genes.

### DD1

In stage 4 embryos, *DD1 *expression was up-regulated approximately 1.9-fold after exposure to kairomones (Figure 2); among all the genes examined in this study, this was the only gene that responded to kairomones in the embryonic stage. BLAST searches suggested that there were no genes homologous to *DD1 *in other crustaceans and insects. Motif searches using the InterPro database http://www.ebi.ac.uk/Tools/InterProScan/ revealed that *DD1 *has a signal peptide and a dopamine beta-monooxygenase N-terminal (DOMON) domain. DOMON domains are ubiquitous among plants and animals, and exist in a variety of proteins, including dopamine beta-monooxygenase, in which this domain was originally found [50]. In *D. pulex*, *DD1 *is thus considered to be a novel gene containing a DOMON domain. In addition to the aforementioned dopamine beta-monooxygenase (DBM), representative proteins containing a DOMON domain also include tyramine beta-monooxygenase (TBM), which is involved in the biosynthesis of biogenic amines [51]. However, the sequence and the domain structure of *DD1 *were completely different to those of *DBM *and *TBM *(data not shown), and the expression profiles of *DD1 *also did not correspond to those of *DBM *and *TBM *(Table 1, 2, Additional file 1, 2). Consequently, *DD1 *is considered to play a different role than either *DBM *or *TBM*. It is possible that *DD1 *is involved in kairmone reception and/or fate determination in the defense morph, because *DD1 *expression was initiated by the presence of kairomones at embryonic stage 4, which is considered to be a critical period for the reception of kairomones [4-6], before declining over the course of postembryonic development (Figure 2).

### DD2, DD3

In postembryonic first-instars, expression levels of *DD2 *and *DD3 *in the presence of kairomones increased by approximately 3- and 2-fold, respectively (Figure 2). While *DD2 *showed extremely high homology to bacterial ribosomal RNA (Table 2, Additional file 2), the *DD2 *sequence in the *D. pulex *genome database was found to contain introns. Furthermore, the full *DD2 *sequence obtained by RACE-PCR had a 3' poly(A) tail, which is not typically present in bacterial transcripts and suggests that the identification of *DD2 *was not the result of contamination. Interestingly, in addition to kairomone responsiveness, the expression levels of *DD2 *were more than 100-fold higher in the first-instar juveniles than in the embryos (Figure 2). Based on these findings, it is possible that *DD2 *may have been acquired by horizontal transfer from bacteria.

*DD3 *exhibited similarity to growth and transformation-dependent protein (GTD-P) (Table 2, Additional file 2). Although *GTD-P *homologues have been identified in some arthropod species, little is known about its functions. However, it was reported that *GTD-P *was strongly expressed when a rat pheochromocytoma cell line (PC12) was exposed to nerve growth factor (NGF) [52]. As it was reported that PC12 cells exposed to NGF undergo proliferation, it is possible that *GTD-P *is involved in the cellular proliferation observed during defense morph formation in *D. pulex*.

## Conclusion

**Figure 3:**
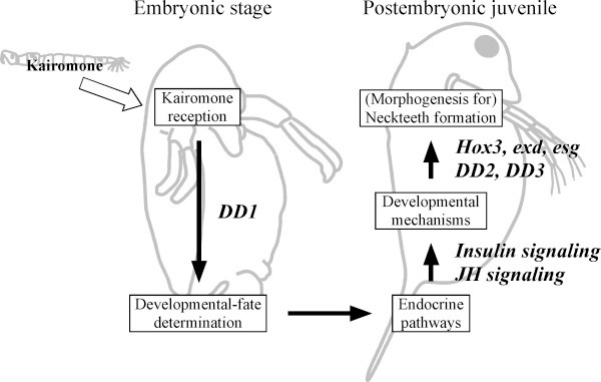
**Schematic diagram showing the process of defense morph formation with the putative involved genes and biological pathways suggested by the present study**. *DD1 *is thought to be involved in kairomone reception and/or fate determination during the embryonic stage. The other genes are considered to be involved in the morphogenesis of postembryonic juveniles.

In this study, we compared the gene expression profiles in the presence/absence of predator kairomones in *D. pulex *embryos and first-instar juveniles. Most of the differences in gene expression induced by the kairomone exposure were observed in postembryonic juveniles, while a single novel gene, *DD1*, was up-regulated in the embryonic stage. Taken together, a putative physiological and developmental cascade for the defense morph formation consisting of the following steps is suggested: 1) Kairomone reception by embryos, 2) Physiological changes through endocrine mechanisms including JH and insulin pathways, 3) Morphogenesis triggered by pattern formation genes (Figure 3).

Based on the results obtained in this study, many genes are thought to be involved in the *D. pulex *defense morph formation. Although we discussed about these genes exclusively in the developmental contexts, it is also possible that they contribute to the plasticity in life history traits such as depth selection [53]. Furthermore, we might underestimate gene expression levels in the kairmone-exposed first-instar juveniles because they were protected in mothers' brood chamber during their sensitive period. For these reasons, given that other genes not identified in this study may also be involved in the predator-induced polyphenisms, further analyses deserve to be undertaken to identify such genes. Furthermore, for the genes identified in this study, localization and functional analyses need to be performed to further clarify the mechanism of inducible defense in *D. pulex*.

## Methods

### Animals

The *Daphnia pulex *clone used in the experiments was provided by the National Institute for Environmental Studies (NIES), Tsukuba, Japan. The NIES clone, originally from Lake Kasumigaura in Japan, was reared in the laboratory at 20°C in aged tap water and fed unicellular green algae (Chlorella Industry Co. Ltd, Fukuoka, Japan) over generations. Using an established rearing method [54], populations of the *Daphnia *clone were maintained in 1 L beakers in a temperature- and photocycle-controlled incubator (20°C, 16-h light/8-h dark).

Fourth-instar *Chaoborus flavicans *larvae were collected from a pond at NIES and maintained in dechlorinated tap-water at a density of 10-15 larvae/L for more than 7 days in a temperature- and photocycle-controlled incubator on a diet of *D. pulex*.

### Kairomone medium

After incubation with *Chaoborus *larvae, the water was filtered using a Whatman GF/C filter (Whatman, London, UK) to remove any daphnid juveniles and particles larger than approximately 1 μm, before being stored in plastic bottles at -20°C. For the experiments, the frozen water was thawed in an incubator (23°C) and used as a rearing medium for *D. pulex *(*Chaoborus*-conditioned medium). Dechlorinated tap-water, in which *Chaoborus *had never been reared, was filtered using a Whatman GF/C filter for use as control medium. The experimental and control media were changed every day.

### Induction of defensive morph and total RNA extraction

Stage 4 embryos and postembryonic first instar juveniles of *D. pulex *were severally used to detect genes specifically expressed in response to the exposure to predator kairomones, with particular emphasis on the expression of those genes that manifested in defense morph formation. We focused on these developmental stages, because, after exposure to kairomones during the embryonic stages of development, fate determination into the defense morph should occur during embryonic stage 4 and morphogenesis of the crest and neckteeth should occur in the first instar (Figure 1B).

To obtain total RNA from stage 4 embryos, very early stage embryos (stage 1-2) were removed from the maternal brood chambers and treated with either the kairomone or control media. After incubation at 20°C for 24-48 h, stage 4 embryos were collected and total RNA was extracted from about 100 individuals for each treatment (kairomone or control), using RNAqueous^®^-Micro (Ambion, Austin, TX, USA). To obtain total RNA from first-instar juveniles, egg-bearing females were reared in the kairomone and control media (20 individuals/500 ml-beaker of adult female *D. pulex*: 1 × 10^6^cells/ml *of Chlorella *for food). After one-week of rearing, newly-born juveniles were harvested and continuously incubated in the kairomone media. From the next day onward, only first-instar juveniles were collected everyday. Harvested individuals were then frozen in liquid nitrogen and preserved at -80°C. Total RNA was extracted from approximately 2,000 daphnia for each treatment (kairomone and control) using an SV Total RNA Isolation System (Promega, Madison, WI, USA). To ensure reproducibility, we conducted an additional series of induction experiments and repeated the RNA extraction procedure for quantitative RT-PCR. In addition, to control for any possible artifacts due to freezing of the kairomone medium or any elution from the plastic bottles used for sample storage, we also tested individuals that had been exposed to control medium that had been frozen and melted in the plastic bottles.

### Candidate gene approach

Amino acid sequences of arthropod gene homologues that were thought to be involved in *Daphnia *defense morph formation (such as *Hox *genes, morphogenetic genes, endocrine genes and neuronal genes) were obtained using euGene's Arthropod genomes http://insects.eugenes.org/arthropods/, and aligned by the CLUSTALW program from GenomeNet http://align.genome.jp/. Amino acid alignments were made principally with the flour beetle (*Tribolium castaneum*), the pea aphid (*Acyrthosiphon pisum*), several species of *Drosophila*, and the tick (*Ixodes scapularis*). Based on the aligned sequences, conserved regions were identified and used to perform TBLASTN searches against the wFleaBase http://wfleabase.org/ to identify *D. pulex *homologues. Subsequently, the predicted coding sequences generated by gene prediction software on wFleaBase (Gnomon, Dappu v1.1 genes, SNAP gene predictor) were used as the sequences for the candidate genes (Table 1, Additional file 1). Furthermore, to improve reliability, these sequences were used as queries to perform BLASTX searches on the National Center for Biotechnology Information (NCBI) server http://blast.ncbi.nlm.nih.gov/Blast.cgi. These analyses helped us to confirm the true homologues of *D. pulex *(Table 1, Additional file 1).

### Differential display

Differential display (DD) was employed to identify genes that were involved in defense morph formation, but which were not detected using the candidate gene approach. The DD assays were performed according to a previously-described method with slight modifications [19,55,56], using the total RNA of stage 4 embryos and first-instar juveniles exposed to the kairomone and control media. Briefly, first-strand cDNA was synthesized from DNase-treated total RNA (500 ng) using SuperScript III (Invitrogen, Carlsbad, CA) and oligo-dT anchor primer (5'-CCC GGA TCC T_15 _G-3'). The resultant cDNA samples were amplified by PCR in reaction mixtures (20 μl) containing 20 combinations of arbitrary 12-mers with a *Hin*dIII site (*Hin*dIII-1 to -20 primers, 5'-CGG GAA GCT TN_12_-3', where N is any base, 4 μM), the anchor primer (20 μM), and AmpliTaq Gold polymerase (0.5 units, Applied Biosystems, Inc., Foster City, CA). The PCR conditions were as follows: one cycle at 94°C for 5 min, followed by 40 cycles at 94°C for 30 sec, 40°C for 2 min and 72°C for 30 sec, and a final extension at 72°C for 5 min. The PCR products were separated on a non-denaturing 12% polyacrylamide gel and visualized by ethidium bromide staining to identify differential bands between the two experimental conditions. To ensure that the results were reproducible, duplicate PCRs and electrophoresis runs were performed.

### Subcloning and sequencing

Differential cDNA bands were excised from the gels and re-amplified by PCR. Subsequently, the cDNA fragments were cloned into pGEM-T vector (Promega) according to the manufacturer's instructions before being sequenced with Big Dye terminator kit on a Model 3100 DNA sequencer (Applied Biosystems). To identify homologous sequences and to estimate gene function, similarity searches were performed with obtained sequences using the wFleaBase, NCBI BLAST database and the European Bioinformatics Institute (EBI) InterPro database http://www.ebi.ac.uk/Tools/InterProScan/.

Since the homologous gene was not found using the gene predictor for the obtained *DD2 *fragment, rapid amplification of cDNA ends (RACE)-PCR was performed using synthesized RACE primers (5'-CCGTTACTCTTTAGGAGGAGACCGCCCC-3' for the 5'-RACE and 5'-TAGGATAGGTGGGAGGCTTTGAAGCGGG-3' for the 3'-RACE) and the SMART RACE cDNA Amplification Kit (Clontech, Palo Alto, CA) to obtain the full-length sequence of *DD2*. The amplified cDNA fragments were cloned and sequenced as described above, and the full-length sequence thus obtained was subjected to an NCBI BLAST search.

### Real-time quantitative RT-PCR

Among the identified genes obtained by the candidate gene approach and by the differential display, the relative expression levels associated with the two experimental conditions (kairomone and control) were quantified and compared using real-time quantitative RT-PCR. Total RNAs of stage 4 embryos and first-instar juveniles that had been exposed to the kairomone and control media were extracted and reverse-transcribed using the conditions described above, except that random hexamer primers were used instead of the oligo-dT primer. Relative quantification of cDNAs was performed using a SYBR Green I chemistry system and ABI Prism 7500 sequence detection system (Applied Biosystems). As endogenous controls of constitutive expression, GAPDH (glyceraldehyde-3-phosphate dehydrogenase) (Accession No. FJ668125), actin (Accession no. AJ245732) and/or 18S ribosomal RNA (Accession no. AF014011) genes were used. Primers for both target and control genes were designed using Primer Express software (Applied Biosystems, see Additional file 3). Data acquisition and analysis were performed by ABI Prism 7500 SDS software ver. 2.0.1 (Applied Biosystems). The baseline and threshold for the Ct (cycle threshold) were set automatically. Each gene was tested in triplicate and standard errors were calculated by the relative standard curve method as described in User Bulletin 2 for the ABI Prism 7700 Sequence Detection System (Applied Biosystems). Statistical analysis was performed based on [57].

## Authors' contributions

HM, MI and TM designed the experiments; HM, MI, NS, YI, AI, HI, YO, SM, SK and RC performed the experiments; HM, MI, YI, AI, HI, SK and TM analyzed the data; HM, MI and TM wrote the paper. All authors have read and approved the final manuscript.

## Supplementary Material

Additional file 1 Table S1: Annotations and expression profiles of investigated candidate genes. Click here for file

Additional file 2 Table S2: Annotations and expression profiles of the genes obtained by differential display (DD). Click here for file

Additional file 3 Table S3: Protein ID and primers for real-time qPCR. Click here for file
